# New Insight into the Molecular Mechanism of the *FUT2* Regulating *Escherichia coli* F18 Resistance in Weaned Piglets

**DOI:** 10.3390/ijms19113301

**Published:** 2018-10-24

**Authors:** Zhengchang Wu, Haiyue Feng, Yue Cao, Yanjie Huang, Chaohui Dai, Shenglong Wu, Wenbin Bao

**Affiliations:** 1 Key Laboratory for Animal Genetics, Breeding, Reproduction and Molecular Design of Jiangsu Province, College of Animal Science and Technology, Yangzhou University, Yangzhou 225009, China; zcwu@yzu.edu.cn (Z.W.); hyfeng24@gmail.com (H.F.); cy457873057@gmail.com (Y.C.); y1426750032@gmail.com (Y.H.); jinghostwj@gmail.com (C.D.); slwu@yzu.edu.cn (S.W.); 2 Joint International Research Laboratory of Agriculture & Agri-Product Safety, Yangzhou University, Yangzhou 225009, China

**Keywords:** pig, *Escherichia coli* F18, *FUT2*, expression, methylation

## Abstract

*Escherichia coli* (*E. coli*) F18 is the main pathogen responsible for post-weaning diarrhea (PWD) in piglets. Resistance to *E. coli* F18 depends on the expression of the cognate receptors in the intestinal epithelial cells. However, the molecular mechanism of *E. coli* F18 resistance in weaned piglets remains unclear. Here, we performed a comparative transcriptome study of the duodenal tissue from Sutai *E. coli* F18 sensitive and resistant pigs by RNA-seq, and pig α(1,2) fucosyltransferase 2 (*FUT2*) was identified as a host differentially expressed gene controlling the *E. coli* F18 infection. Function analysis showed that the *FUT2* expression was high in the duodenum and jejunum, with higher levels detected in sensitive individuals than in resistant individuals (*p* < 0.01). Expression levels of *FUT2* were upregulated in IPEC-J2 cells after lipopolysaccharide (LPS)-induction or *E. coli* stimulation. *FUT2* knockdown decreased the adhesion of *E. coli* F18 to IPEC-J2 cells (*p* < 0.05). *FUT2* overexpression markedly increased the adhesion of *E. coli* F18 to IPEC-J2 cells (*p* < 0.05 or *p* < 0.01). Furthermore, the *FUT2* mRNA levels correlated with methylation levels of the mC-22 site in the specificity protein 1 (Sp1) transcription factor (*p* < 0.05). Electrophoretic mobility shift assays (EMSA) showed that Sp1 interacts with the wild-type *FUT2* promoter DNA, but not with methylated DNA. Our data suggested that *FUT2* methylation at the mC-22 site inhibits Sp1 binding to the *FUT2* promoter, thereby reducing *FUT2* expression and enhancing *E. coli* F18 resistance in weaned piglets. These observations highlight *FUT2* as a promising new target for combating *E. coli* F18 susceptibility in weaned piglets.

## 1. Introduction

Porcine post-weaning diarrhea (PWD) causes serious economic losses to large-scale pig farms. Enterotoxigenic *Escherichia coli* F18 (ETEC18), which is one of the major pathogens responsible for PWD, adheres to the small intestinal epithelial cells of piglets through its pili. The subsequent binding to the brush border F18 receptors of these cells leads to the enterotoxin production that causes diarrhea in piglets. Hence, the pathogenicity of *E. coli* F18 depends on the expression of the corresponding receptors by the brush border of the piglet small intestinal epithelial cells [1]. Recently, with the development of high-throughput next-generation sequencing (NGS) technologies, transcriptome profiling by RNA sequencing (RNA-seq) can now be used to provide novel insights into molecular mechanisms [2]. To obtain individuals with an extreme phenotype for RNA sequencing, our research team previously established the Sutai pig (a new hybrid between the Duroc and Meishan breeds), a population that includes both *E. coli* F18-resistant and -sensitive individuals [3]. In this study, we performed a comparative transcriptome study of porcine duodenum tissue in Sutai *E. coli* F18-sensitive and -resistant pigs using RNA-seq. Furthermore, we identified the differential expression of the *FUT2*, *FUT3*, *TLR5*, *TAP2*, *IL1β* genes in the duodenum, indicating that these genes probably play a crucial role in the resistance to *E. coli* F18. Our previous studies concentrated on the relationship of immune gene expression and *E. coli* F18 resistance in pigs [4,5,6,7,8], only a few reports directly on receptor formation showing the effect of the α(1,2) fucosyltransferase 1 (*FUT1*) gene M307 G/A mutation on the adhesion of *E. coli* F18 to pig intestinal epithelial cells [9], but its polymorphism distribution in more than 20 Chinese local pig breeds and wild boar population is extremely skewed [10,11,12]. The smallest antigenic determinant of the *E. coli* F18 receptor is the type 1 H-antigen of the ABO blood group antigens [13]. ABO Blood group antigens are mainly distributed in red blood cells, secretions, and some tissues. ABO expression is regulated by two α(1,2)fucosyltransferases (FUT1, FUT2) [14,15]. Here, we hypothesized that the *FUT2* gene (but not the *FUT1* gene) directly catalyzes the formation of the *E. coli* F18 receptor in pigs. To explore the relationship between the *FUT2* gene expression and *E. coli* F18 resistance in weaned piglets, we used the qPCR and western blot analyses to investigate whether the *FUT2* expression correlated with *E. coli* F18 resistance in an LPS-induced or bacteria-stimulated small intestinal epithelial cell line (IPEC-J2), as well as in intestinal tissues of Sutai sensitive and resistant pigs. We also performed a functional analysis of the *E. coli* F18 adhesion in vitro using RNA interference and overexpression.

DNA methylation is a basic element of epigenetic modification that does not change the base sequence or composition. Nevertheless, methylation of promoter region cytosines tends to inhibit gene transcription and is associated with some diseases [16,17,18]. Furthermore, DNA methylation profiles are also tissue-specific and developmental stage-specific [19,20,21]. Conventional methods used for methylation quantification include sanger sequencing and pyrosequencing. Sanger sequencing has shortcomings such as a low quantitative accuracy, heavy workload and low time efficiency because of the limited number of selectable clones and sample disparities between clones selected from different batches [22]. Pyrosequencing quantifies the degree of methylation by detecting fluorescence, which thus also has the disadvantage of low accuracy, especially in the hypermethylation or hypomethylation states. Furthermore, the relative reading sequence is short in pyrosequencing and generally does not exceed 100 bp, which does not cover the complete CpG island region [23]. With the continuous research on DNA methylation and the development of gene sequencing technology, a novel method termed bisulfite amplicon sequencing (BSAS) can generate sequencing reads up to 2 × 300 bp in length, which allows for the coverage of most CpG islands [24]. In this study, we used bisulfite amplicon sequencing (BSAS) to determine the methylation levels of CpG islands in the *FUT2* promoter region in intestinal tissues of weaned piglets. Furthermore, we analyzed the effects of the important methylation site on mRNA expression and the key transcription factors in the promoter region, and further verified key transcription factors using electrophoretic mobility shift assay (EMSA) and in vitro methylation. This study not only revealed the regulatory mechanism of *FUT2* expression controlling *E. coli* F18 resistance in weaned piglets, but also provided the basis of strategies for the bioengineering regulation of *E. coli* F18 resistance in human and pigs.

## 2. Results

### 2.1. FUT2 Was Identified as a Host Gene Related to E. coli F18 Resistance Based on an RNA-Seq Analysis

**Figure 1:**
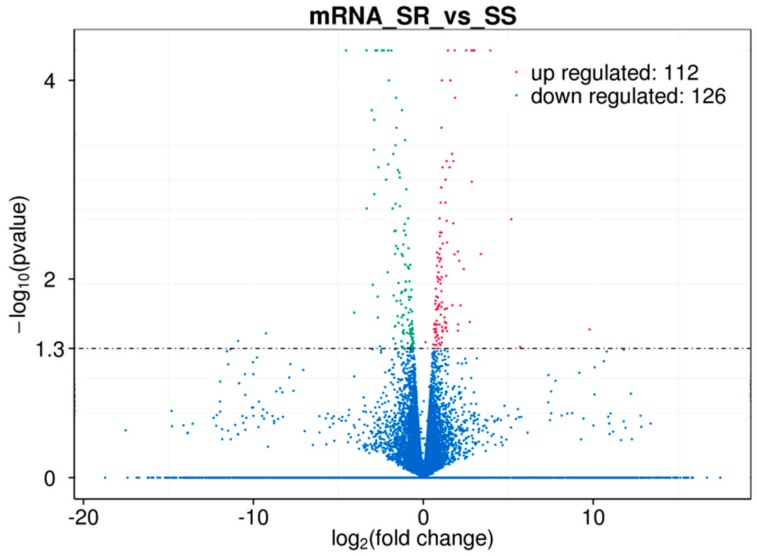
A volcano plot displaying the differentially expressed genes (DEGs) between the *E. coli* F18-resistant (*n* = 3) and -sensitive groups (*n* = 3) based on RNA-seq analysis. Red dots represent upregulated DEGs; green dots represent downregulated DEGs; blue dots represent non-DEGs; dotted line represents a screening threshold for DEGs; the *x*-axis values correspond to the log_2_ (fold change) value; the *y*-axis corresponds to the mean expression value of the −log_10_ (*p*-value) between the *E. coli* F18 sensitive (SS) and *E. coli* F18 resistant (SR) groups (fold change = SR/SS).

**Figure 2:**
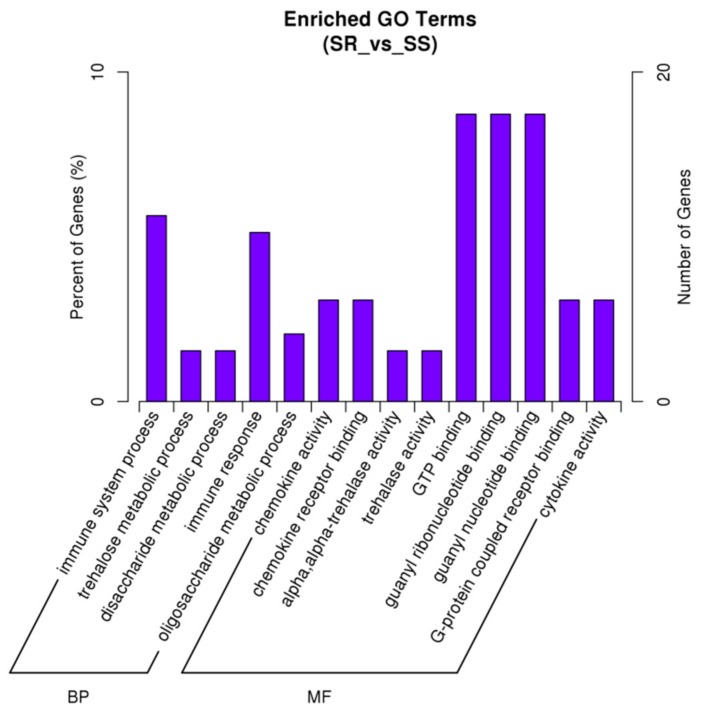
Gene ontology (GO) enrichment. The *x*-axis shows the second level GO terms from the biological process (BP) and molecular function (MF) while the *y*-axis shows the number and percentage of gene enrichment.

**Figure 3:**
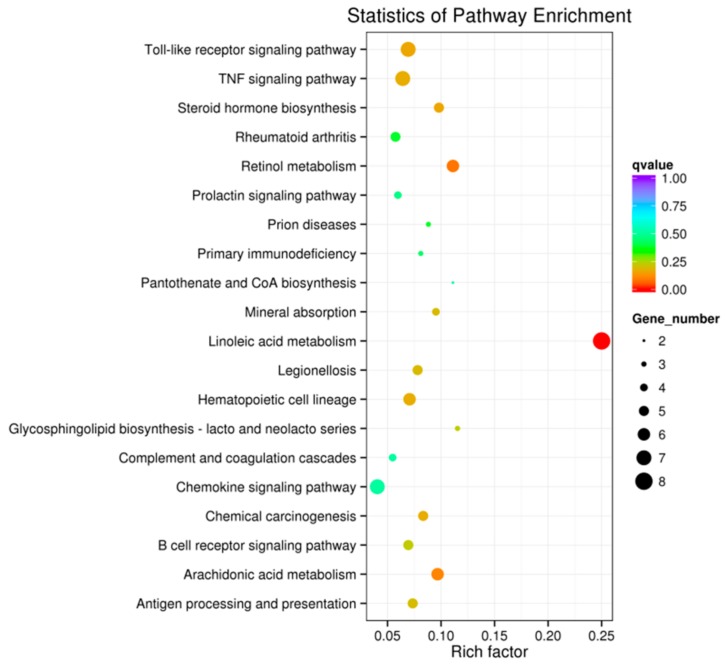
The Kyoto Encyclopedia of Genes and Genomes (KEGG) pathway analysis of differentially expressed genes (DEGs), with the *x*-axis showing an enrichment factor and the *y*-axis showing the pathway name; the point size represents the number of DEGs and the point color represents the *q*-value range.

We performed a comparative transcriptome sequencing analysis of duodenal tissues from *E. coli* F18-resistant (SR, *n* = 3) and sensitive piglets (SS, *n* = 3) (data submitted to NCBI’s SRA repository BioProject ID: PRJNA476721, PRJNA476722). We obtained 132.46, 140.51, 132.18, 146.49, 137.19, and 126.64 million raw reads for SR1, SR2, SR3, SS1, SS2, and SS3, respectively. We performed sequence alignment against the reference pig genome (Sscrofa10.2, ftp://ftp.ensembl.org/pub/release-87/fasta/sus_scrofa/dna/) using MapStat. After quality control, approximately 82.0% (81.2–82.7%) of reads were mapped to the reference genome. Of these, 68.5–71.8% mapped uniquely to the pig reference genome, and 9.4–13.5% showed multiple matches. To further clarify the molecular mechanism by why resistance to *E. coli* F18 infection is regulated, we identified 238 DEGs between *E. coli* F18-resistant and sensitive piglets according to the principle that *p* < 0.05 and |log_2_(fold change)|< 1. Of these, 112 DEGs were upregulated in the *E. coli* F18-resistant group compared with the sensitive group (Figure 1, Table S2). Further function annotations of transcripts were shown in Figure 2, Table S3 and in Figure 3, Table S4. We identified some important pathways and DEGs, including immune pathways (“Toll-like receptor signaling pathway” (*TLR5*, *IL1β*) and “Antigen processing and presentation” (*TAP2*)) and a glycolipid-synthesis pathway (“Glycosphingolipid biosynthesis-lacto and neolacto series” (*FUT2*, *FUT3*)). The previous study showed that the *FUT1* gene M307 G/A mutation can affect the adhesion of *E. coli* F18 to pig intestinal epithelial cells [9]. On this basis, we selected some important genes (*TLR5*, *IL1β*, *FUT1*, *FUT2*, *FUT3*) for qRT-PCR validation. The results showed (Figure S1) that the expression levels of *FUT2* and *FUT3* were extremely significantly higher in the *E. coli* F18-sensitive group than that in the *E. coli* F18-resistant group (*p* < 0.01); the expression levels of *TLR5*, *IL1β*, and *TAP2* were significantly higher in the *E. coli* F18-sensitive group than that in the *E. coli* F18-resistant group (*p* < 0.05). However, *FUT1* had no significant difference between the *E. coli* F18-sensitive group and the -resistant group (*p* > 0.05). Meijerink et al., 1997 demonstrated that the synthesis of the *E. coli* F18 FedF adhesion protein was in association with *FUT1* and *FUT2* [14,15]. Therefore, this study focused on the *FUT2* gene for further in-depth analysis.

### 2.2. Decreased Expression of FUT2 Contributes to Enhancing E. coli F18 Resistance in Piglets

**Figure 4:**
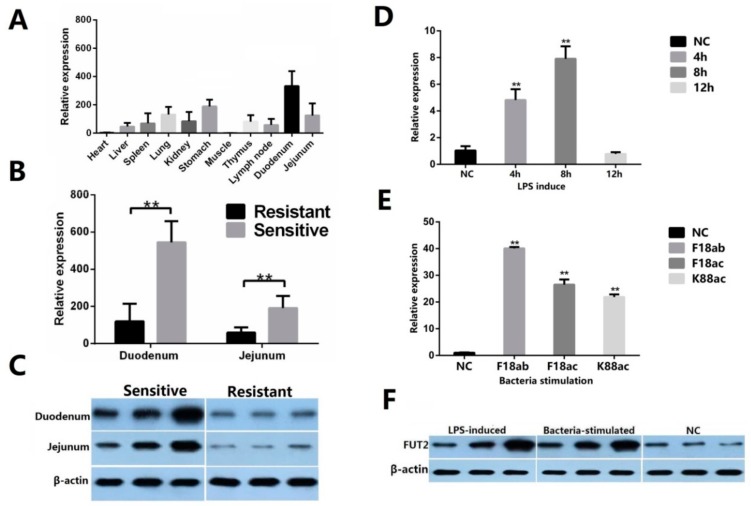
The association between *FUT2* expression and the *E. coli* F18 infection. (**A**) mRNA expression profile in different tissues; data are shown as mean ± SD; (**B**) qRT-PCR analysis of differential expression in intestinal tissues between *E. coli* F18 resistant and sensitive piglets, data are shown as mean ± SD, *n* = 3 biological replicates, ** *p* < 0.01; (**C**) western blot analysis of differential expression in intestinal tissues between *E. coli* F18 resistant and sensitive piglets, *n* = 3 biological replicates, β-actin, internal reference; (**D**–**F**) Expression validation of important DEGs in IPEC-J2 cells after lipopolysaccharide (LPS)-induction and bacterial stimulation. IPEC-J2 cells were stimulated with 0.1 μg/mL LPS or *E. coli* F18ab, *E. coli* F18ac, and *E. coli* K88ac; (**D**,**E**) qRT-PCR analysis, data are shown as mean ± SD, *n* = 3 biological replicates, ** *p* < 0.01, NC: negative control; (**F**) Western blot analysis. β-actin, internal reference; NC: negative control, LPS-induced represents the cells induced with 0.1 μg/mL LPS for 4, 8, and 12 h, respectively; bacteria-stimulated represents the cells stimulated with *E. coli* F18ab, *E. coli* F18ac, and *E. coli* K88ac, respectively, *n* = 1 biological replicate.

Fluorescence qPCR revealed distinct *FUT2* gene expression profiles in different tissues of Sutai piglets, with a relatively high expression detected in the duodenum and jejunum (Figure 4A). To further investigate the relationship between *FUT2* expression and *E. coli* F18 infection, using a quantitative real-time polymerase chain reaction (qRT-PCR) (Figure 4B) and western blot analysis (Figure 4C), differential expression validation showed that *FUT2* gene expression levels in the duodenum and jejunum of resistant individuals were remarkably lower than those in sensitive individuals (*p* < 0.01). IPEC-J2 cells were exposed to LPS (0.1 μg/mL) for 4, 8, and 12 h, as well as to the *E. coli* F18ab, F18ac, and K88ac strains (10^9^ CFU/mL). qRT-PCR analysis showed that *FUT2* expression was increased at 4 and 8 h (*p* < 0.05) (Figure 4D) or in *E. coli* strain-stimulated IPEC-J2 cells (*p* < 0.01) (Figure 4E). Western blot analysis confirmed that the expression of these genes is upregulated in IPEC-J2 cells following LPS-induction and *E. coli* strain stimulation (Figure 4F).

**Figure 5:**
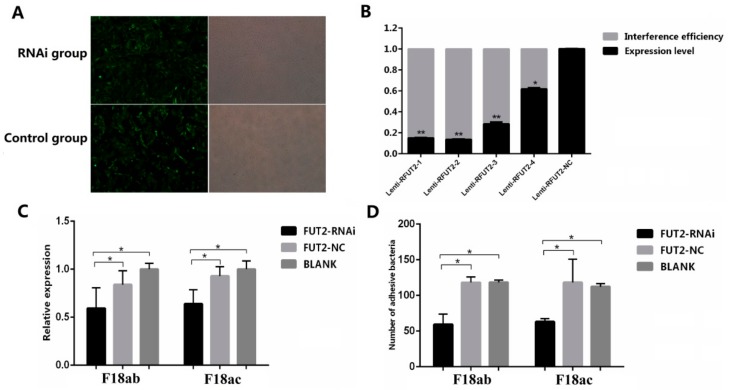
The effect of the *FUT2* gene knockdown on the adhesion of *E. coli* F18 to IPEC-J2 cells. (**A**) Green fluorescence protein (GFP) expression recorded under a fluorescence microscope (100×); (**B**) Interference efficiency evaluation by detecting mRNA expression levels of *FUT2* in non-treated (BLANK) cells and those transfected with lentiviruses containing non-silencing small hairpin RNA (*FUT2*-NC) and *FUT2*-RNAi determined by qRT-PCR analysis, data are shown as mean ± SD, *n* = 3 biological replicates, ** *p* < 0.01, * *p* < 0.05; (**C**,**D**) Adhesion of the F18 fimbria to IPEC-J2 cells analyzed by relative quantification (**C**) and bacteria enumeration (**D**) detection, data are shown as mean ± SD, *n* = 3 biological replicates, * *p* < 0.05.

**Figure 6:**
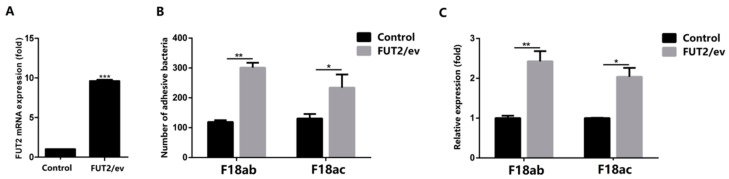
The effect of *FUT2* gene overexpression on the adhesion of *E. coli* F18 to IPEC-J2 cells. (**A**) Overexpression efficiency evaluation by detecting mRNA expression levels of *FUT2* in pcDNA3.1 (Control) cells and pcDNA3.1-FUT2 (FUT2/ev) cells, *** *p* < 0.001; (**B**,**C**) Adhesion of the F18 fimbria to IPEC-J2 cells analyzed by bacteria enumeration (**B**) and detection relative quantification (**C**), data are shown as mean ± SD, *n* = 3 biological replicates, * *p* < 0.05. ** *p* < 0.01.

Besides, we used lentivirus-mediated RNAi to mediate the specific suppression of *FUT2* in the pig epithelial cell line IPEC-J2. As shown in Figure 5A, a green fluorescence protein expression was detected in more than 90% of shRNA-treated cells. The knockdown efficiency of *FUT2* in Lenti-R*FUT2*-n (*n* = 1, 2, 3, 4, NC) treated IPEC-J2 cells compared with non-treated cells was 85.1%, 86.5%, 71.6%, and 38.3%, respectively, and Lenti-R*FUT2*-2 treated IPEC-J2 cells were used for the subsequent investigations (Figure 5B). Relative quantification (Figure 5C) and bacteria enumeration (Figure 5D) showed a reduced adhesion of *E. coli* F18 to IPEC-J2 cells treated with *FUT2*-RNAi via F18ab/ac-expressing fimbriae compared with that of non-treated and Lenti-R*FUT2*-NC treated cells (*p* < 0.01). As shown in Figure 6A, the expression of *FUT2* in IPEC-J2 cells with pcDNA3.1-FUT2 increased to 9.6 times. Bacteria enumeration (Figure 6B) and relative quantification (Figure 6C) showed the markedly increased adhesion of *E. coli* F18 to IPEC-J2 cells treated with pcDNA3.1-*FUT2* via F18ab/ac-expressing fimbriae compared with that of pcDNA3.1 treated cells (*p* < 0.05 or *p* < 0.01). Together, all these results suggested that the decreased expression of *FUT2* contributes to enhancing *E. coli* F18 resistance in piglets.

### 2.3. Effect of FUT2 Promoter Methylation Level on Gene Expression

**Figure 7:**
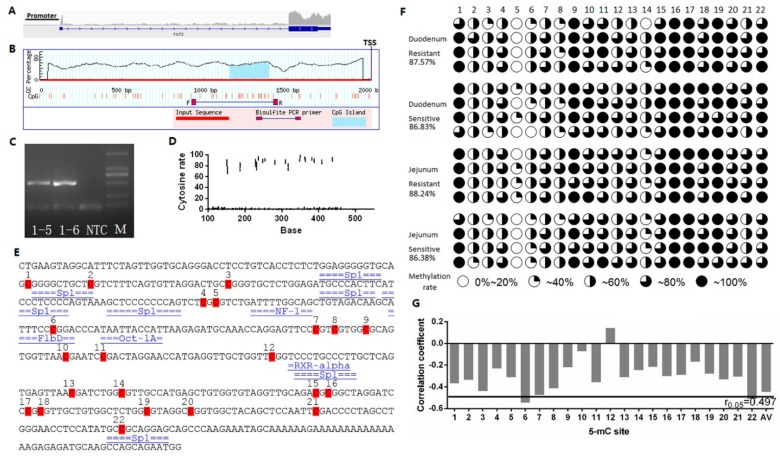
The effect of the *FUT2* promoter methylation level on gene expression (**A**) *FUT2* gene promoter analysis of transcriptome sequencing results through Integrative Genomics Viewer digging. Different peaks represent the differences in the expression quantity of different exon regions; the long blue bar represents the pig genome sequence information in the database; gray bars indicate the transcriptome sequencing reads after stitching; TSS: transcriptional start site; (**B**) Detection of the *FUT2* gene promoter CpG islands; (**C**) Agarose gel electrophoresis of *FUT2* methylation primer amplification products, Lanes 1–5, 1–6: template PCR product; NTC: no template control PCR product; M: DL2000 DNA Marker; (**D**) Cytosine ratio of the amplified fragments; (**E**) Sequence analysis of *FUT2* gene methylation detection; (**F**) Methylation level of the *FUT2* gene amplified fragment in the duodenum and jejunum; Red box represents CpG sites; (**G**) Correlation analysis between the *FUT2* gene amplified fragment methylation and mRNA expression level, AV: the average degree of methylation.

Based on an integrative genomics viewer (IGV) analysis of transcriptome sequencing (Figure 7A), we obtained the 2000 bp sequence upstream of the *FUT2* gene. MethPrimer analysis of the 2 Kb upstream region of the *FUT2* gene revealed a single CpG island (Figure 7B). PCR amplification products were examined using 1% agarose gel electrophoresis and its fragment size was consistent with the expected product (506 bp), and each amplification yielded a single specific product that was cloned directly for sequencing (Figure 7C).

Using bisulfite amplicon sequencing (BSAS), we performed the methylation analysis of the *FUT2* promoter in intestinal tissues between *E. coli* F18-sensitive and -resistant individuals (Figure 4). As shown in Figure 7D, we plotted the distribution of the overall cytosine percentages. 22 CpG sites were detected in the PCR amplicon of pig *FUT2* promoter, and the average methylation levels ranged from 86.38% to 88.24% in the duodenum and jejunum tissues (Figure 7F). Moreover, we presented the binding sites of transcription factors located within CpG sites in the core promoter region of the *FUT2* gene, including Sp1, NF-1, F1bD, Oct-1A, and RXR-alpha (Figure 7E). Pearson correlation analysis showed that the methylation level of the CpG island was negatively correlated with the *FUT2* mRNA expression (Figure 7G, *R* = −0.467, *r*_0.05_ = 0.497, where *r*_0.05_ is the correlation coefficient threshold), with significant correlation coefficients being obtained only for the mC-6 and mC-22 sites (*R* = −0.42). Furthermore, mC-6 was located in F1bD and mC-22 was located in the Sp1 (specificity protein 1) transcription factor binding site (TFBS). Studies showed the methylation of some CpG sites could decrease the efficiency of binding between the transcription factors and promoters, thereby lowering the gene transcription rate. Sp1 belongs to the methylation-dependent transcription factors and Sp1 binding to DNA has a transcription-activating effect [25,26,27]. Therefore, these data indicated that *FUT2* methylation at the mC-22 site suppresses the *FUT2* expression.

### 2.4. EMSA Analysis of Sp1 Binding to the FUT2 Gene Promoter

**Figure 8:**
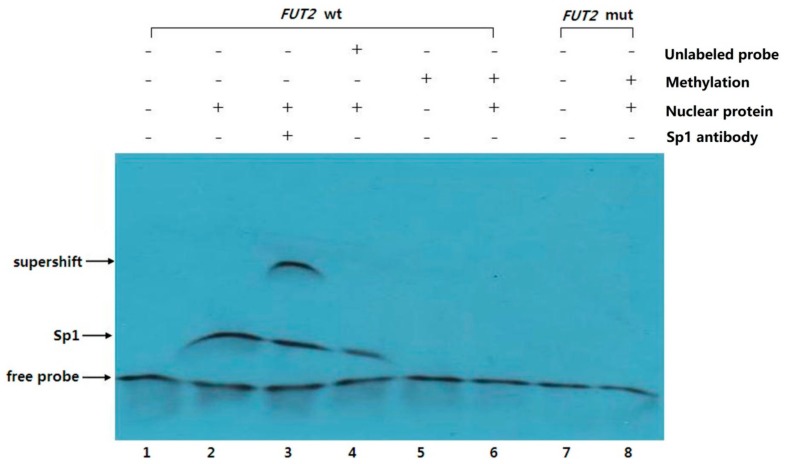
The electrophoretic mobility shift assay (EMSA) analysis of Sp1 binding to the *FUT2* promoter. Nuclear proteins from duodenal tissues expressing Sp1 were extracted using the nuclear protein extraction kit. All products were electrophoresed on a non-denaturing 5% polyacrylamide gel. After electrophoresis, DNAs were transferred to a positive nylon membrane, UV cross-linked, probed with a streptavidin-HRP conjugate and incubated with the substrates of the ECL kit. All free biotin-labeled probes (*FUT2* wt, Lanes 1–6; *FUT2* mut, Lanes 7 and 8) were detected by streptavidin-HRP. The Add unlabeled probe in Lane 4 was a cold competitor. Probes in Lanes 5 and 6 and Lane 8 were treated by CpG methyl transferase *M. SssI*. Add Sp1 antibody, a strong supershifted band was detected in Lane 3.

The effect of *FUT2* methylation on Sp1 binding to the *FUT2* promoter was investigated using EMSA (Figure 8). Several shift bands were observed with unmethylated *FUT2* wild-type probes (Lanes 2–4), while none were detected with methylated wild-type (Lane 6) and mutant-type (Lane 8) probes. These observations confirmed that *FUT2* wild-type probes bind with nuclear extracts from duodenal tissues. Furthermore, in the presence of the Sp1 antibody, a strong supershifted band was observed with the unmethylated wild-type probe only (Lane 3), providing further evidence that Sp1 from nuclear extracts binds specifically to the unmethylated *FUT2* wild-type probe. The above results indicated that *FUT2* methylation at the mC-22 site inhibits Sp1 binding to the *FUT2* promoter.

## 3. Discussion

Post-weaning diarrhea (PWD) is caused by enterotoxin production resulting from *E. coli* F18 adherence to the small intestinal epithelial cells of piglets through its fimbriae and binding to the F18 receptors in the brush border of these cells. Thus, the pathogenicity of *E. coli* F18 depends on the expression of these cognate receptors [28]. In a study of 74 Landrace pigs at 0–23 weeks of age, Coddens et al., 2007 found that the *E. coli* F18 receptor expression was elevated with increasing age, with gradually increased levels detected during the first three weeks and stably maintained levels detected from 3–23 weeks [29]. Unweaned piglets were insensitive to *E. coli* not only because the receptors were yet to be formed in the intestinal tract, but also because the antibodies in breast milk protected the piglets from enteropathogen infections [30]. Accordingly, one-week post-weaned piglets (i.e., 35 days old) were selected for investigation in this study during which both their phenotype and autoimmunity were most sensitive to *E. coli* F18 infections. In this study, we undertook a strict selection of *E. coli* F18-resistant and -sensitive post-weaned piglets for transcriptome analysis. We identified 238 DEGs probably related to *E. coli* F18 resistance, including *FUT2*. *E. coli* F18 resistance mainly depends on the expression of receptors on intestinal epithelial cells. Studies have shown that the smallest antigenic determinant of the *E. coli* F18 receptor is the type 1 H-antigen of the ABO blood group antigens [13]. Type 1 H-antigen synthesis is catalyzed by the protein encoded by the α-(1,2) fucosyltransferase gene 2 (*FUT2*) [14,15]. These proteins are known as the histo-blood group antigens, which are distributed mainly in the secretion fluids and tissues of ectodermal or mesodermal origin, rather than on the erythrocyte membrane surface [31]. The high *FUT2* gene expression in the intestinal tract and other ectodermal tissues also illustrates this point. In this study, we found a lower *FUT2* expression in lower intestinal tissues of resistant individuals compared with that in sensitive individuals. Meijerink et al., 2000 also reported a very high *FUT2* expression in sensitive individuals, while almost no expression was detected in resistant individuals [9]. In addition, we found that *FUT2* knockdown reduced the ability of *E. coli* F18 to adhere to IPEC-J2 cells. Taken together, these data suggest that the downregulation of *FUT2* expression reduces the *FUT2* enzymatic activity while enhancing resistance to *E. coli* F18.

In this study, the cytosine ratio in the partial *FUT2* gene promoter region was high; therefore, C loci in those genes with an average cytosine ratio exceeding 1% were selected for the analysis of methylation levels in each sample. Although the methylation levels of the *FUT2* gene amplified fragments were relatively conserved, the negative correlation with mRNA levels suggested that CpG island methylation might inhibit *FUT2* gene transcription. Barrera et al., 2012, found that different CpG islands possessed different genomic elements with distinct gene regulation functions [32]. There may be some specific CpG sites affecting CpG island function and methylation [33]. This suggests that despite many CpG sites in each gene promoter region CpG island, not every methylation of the site will change the gene expression [34]. Instead, gene silencing occurs following methylation of only specific sites that regulate gene function. In the current study, methylation rates were accurately determined using single methylation sites as the basic unit (0.01% error). Analyses of single methylation sites in the target fragments revealed a negative correlation in methylation levels between the mC-6 and mC-22 sites of the *FUT2* gene CpG islands (*p* < 0.05), suggesting that these sites might be critical for the regulation of gene transcription, while other methylated sites might be auxiliary.

Combined with the feature analysis of amplified sequences, all CpG sites in the *FUT2* gene amplified fragments were found to be methylated. In terms of the inhibition of expression by DNA methylation, it is generally believed that cytosine C methylation blocks transcription by directly acting on methylation-sensitive transcription factors to destroy their ability to bind DNA binding; it can also act on non-methylation-sensitive transcription factors by attaching themselves to repressor proteins [35]. In this study, mC-22 of the *FUT2* gene was identified on the transcription factor Sp1 binding sites. Studies have shown that CpG sites are present in the specific binding sites of some transcription factors. The methylation of these sites decreases the efficiency of binding between transcription factors and promoters, thereby lowering the gene transcription rate. Such methylation-dependent transcription factors include specificity protein 1 (Sp1) [25,26,27], cAMP-response element binding protein (CREB) [36], myelocytomatosis (Myc) [37,38], upstream transcription factor 1 (USF-1) [39], CCCTC-binding factor (CTCF) [40], GATA binding protein 1 (GATA-1) [41], and APETALA2 (AP-2) [42]. Sp1 binding to DNA has a transcription-activating effect. Multiple copies of its binding sequences are often distributed in gene promoters or enhancers [43,44], and Sp1 regulates gene transcription by binding to the DNA and through the interaction with other proteins.

## 4. Materials and Methods

### 4.1. Experimental Animals

Experimental Sutai pigs were obtained from the *E. coli* F18 resistant and sensitive resource population pre-established by our team (currently maintained at the Sutai Pig Breeding Center in Suzhou, Jiangsu, China). Based on the adhesion assays for *E. coli* F18 binding to pig epithelial cells (IPEC-J2), the previous study has obtained three pairs of F18-resistant and -sensitive individuals with an identical birth weight, weaning weight, body shape, coat color with and feeding in the same environment [45]. During the weaning period, i.e., ~35 days of age, when the piglets are most sensitive to *E. coli* F18 infection and prone to manifestation of diarrheic symptoms, piglets were humanely sacrificed using an intravenous injection of pentobarbital sodium as necessary to ameliorate suffering and collected heart, liver, spleen, lung, kidney, stomach, muscle, thymus, lymph node, jejunum, and duodenum tissues. All tissues were stored in liquid nitrogen in situ and then stored in a −70 °C refrigerator for later use.

The animal study proposal was approved by the Institutional Animal Care and Use Committee (IACUC) of the Yangzhou University Animal Experiments Ethics Committee (permit number: SYXK (Su) IACUC 2012-0029; Approval Date: 09-12-2012). All experimental procedures were performed in accordance with the Regulations for the Administration of Affairs Concerning Experimental Animals approved by the State Council of the People’s Republic of China.

### 4.2. Transcriptome Sequencing and Data Analysis

#### 4.2.1. cDNA Library Preparation and Sequencing

A total amount of 3 μg RNA per sample of duodenum tissues from F18-resistant individuals (*n* = 3, SR) and sensitive individuals (*n* = 3, SS) were used for RNA sample preparation. The total RNA was isolated using TRIzol, after which the purity, concentration, and integrity were determined using a NanoDrop spectrophotometer (IMPLEN, Westlake Village, CA, USA) and a Qubit RNA Assay Kit with the Qubit^®^ 2.0 Fluorometer (Life Technologies, Camarillo, CA, USA). RNA libraries were sequenced using an Illumina HiSeq 2000 Nano 6000 Assay Kit and analyzed with the Bioanalyzer 2100 system (Agilent Technologies, Santa Clara, CA, USA). The libraries were sequenced on an Illumina HiSeq 2000 platform (Novogene Bioinformatics Technology, Beijing, China) and 100 bp paired-end reads were generated.

#### 4.2.2. Identification of Differential Expression Genes (DEGs)

After the measurement of reads per kilobase per million mapped reads (RPKM) value, we performed a differential expression analysis of the transcript between Sutai F18-resistant and -sensitive piglets by Cuffdiff method [46]. A corrected *p*-value < 0.05 and |log_2_(foldchange) < 1| were set as the identification principle of differential expression genes.

#### 4.2.3. Gene Function Annotation

GO (Gene Ontology, http://geneontology.org/) was used for excavating the biological function of differential expression genes, including molecular function, cellular component, and biological process. GO terms were analyzed by the software GOSeq [47], with the principle of corrected *p*-values < 0.05. KEGG (Kyoto Encyclopedia of Genes and Genomes, http://www.kegg.jp/) is a database that could explain the network of molecular interactions in a particular species. Using KOBAS software [48], we performed a KEGG pathways analysis of differential expression genes.

### 4.3. Cell Culture, LPS-Induction and E. coli Stimulation in IPEC-J2 Cells

Pig intestinal epithelial cells (IPEC-J2) offered by the University of Pennsylvania (Philadelphia, PA, USA) were cultured at 37 °C and 5% CO_2_ with DMEM/F12 medium supplemented with 10% fetal bovine serum (FBS). At 80–90% confluence, the cells were exposed to 0.1 μg/mL LPS (Sigma-Aldrich, St. Louis, MO, USA). The cells incubated with culture medium without LPS-induction were regarded as the negative control (NC). Each treated group had three replicates. Cellular RNA and protein were extracted in LPS-induced cells at 4 h, 8 h, and 12 h and control cells, which was used for qRT-PCR and western blot analysis.

*E. coli* F18ab, *E. coli* F18ac, and *E. coli* K88ac (offered by the veterinary laboratory at the Institute of Microbiology, University of Pennsylvania) fimbriae standard strains were inoculated into a Luria–Bertani (LB) culture medium and incubated for 12 h on a rotating shaker table (200 rpm). After centrifugation for 5 min at 4000 rpm, the three types of *E. coli* culture supernatants were filtered (pore size, 0.22 μm) and used to resuspend the bacteria, which were then washed three times.

### 4.4. RNA Interference and Overexpression of FUT2 Gene in IPEC-J2 Cell and its Effects on E. coli F18 Adhesion In Vitro

Four RNAi sequences targeting *FUT2* mRNA (R1: 5′-GTGTAACCACACTGTCATGAC-3′; R2: 5′-GACCATCTACCTGGCCAATTA-3′; R3: 5′-GACTCTCCCTTCCTCAAACTC-3′; and R4: 5′-GGAGCACACATTGTTCCATGT-3′) and one off-target control sequence (NC: 5′-TTCTCCGAACGTGTCACGT-3′) were cloned separately into the LV3-H1/GFP&Puro vector (GenePharma, Shanghai, China) and co-transfected with packaging plasmids into 293T cells (GenePharma). The virus was collected and used to infect the target cells (IPEC-J2). Positive cells were selected by the addition of 10 μg/mL puromycin total RNA, and proteins were extracted from representative cells at 24 h intervals for the analysis of *FUT2* gene expression at the transcriptional and translational levels. The cells infected with R1, R2, R3, or R4 lentivirus *FUT2* shRNA vector were designated Lenti-RFUT2-n (*n* = 1, 2, 3, 4, NC) cells, respectively. Full-length DNA encoding *FUT2* genes (Gene ID: NM_214069) were amplified using the RT-PCR and cloned to pcDNA3.1 vector (Invitrogen, Carlsbad, CA, USA). The primer sequences for cloning the full length of *FUT2* were as follows: F: 5’-AGTTTAAACGGATCTCTAGCGAATTCGCCACCATGCTCAGCATGCAGGCATCTTTCTTC-3’; R: 5’-TCCGGCCTTGCCGGCCTCGAGCGGCCGCTCACTTATCGTCGTCATCCTTGTAATCGTGC-3’. For the IPEC-J2 control, Lenti-RFUT2-n, and pcDNA3.1-FUT2 cells, the in vitro evaluation of F18 fimbriae adhesion was performed as previously described [45]. A relative quantification method [49], in addition to bacteria enumeration, was used to establish an efficient and accurate method for the detection of *E. coli* adhesion to small intestinal epithelial cells in pigs.

### 4.5. qRT-PCR Analysis

Tissues and cells RNA from Sutai pigs were extracted in accordance with the Trizol Reagent instructions (Invitrogen). The RNA integrity was checked by 1% formaldehyde denaturing agarose gel electrophoresis, and the RNA concentration was determined by a ND-1000 nucleic acid/protein concentration tester. cDNA was synthesized by PrimeScript RT-PCR kit (TaKaRa, Beijing, China). Reverse transcription reaction system: 5 × qRT SuperMix II 2 μL, total RNA 500 ng, add RNase free ddH_2_O to make the volume 10 μL. The reaction condition was set as 25 °C for 10 min, 50 °C for 30 min, 85 °C for 5 min, and 4 °C forever. A real-time quantitative PCR instrument ABI7500 (Applied Biosystems, Foster City, CA, USA) was used to conduct qPCR analysis and the amplification program was set as: 95 °C for 30 s; 95 °C for 5 s and 60 °C for 34 s, followed by 40 cycles. The primers of *FUT1*, *FUT2*, *FUT3*, *TLR5*, *IL1β*, *TAP2* amplified fragments and housekeeping gene (*GAPDH*, *β-actin*) were shown in Table S1. Relative quantitative results were analyzed using 2^−ΔΔ*C*t^ method [50].

### 4.6. Western Blot Analysis

The total proteins from tissues and cells were extracted in accordance with the instructions of NE-PER kit (Thermo Fisher Scientific, Waltham, MA, USA). A BCA kit (Nanjing Keygen Biotech, Nanjing, China) was used to normalize protein levels. Proteins were transferred to PVDF membranes, which were then immunoblotted with relevant primary detection antibodies (FUT2 (1:600) and β-actin (1:4000)) followed by a secondary detection antibody (horseradish peroxidase (HRP) conjugated goat anti-rabbit IgG (Abcam, Cambridge, UK, 1:5000)).

### 4.7. Bioinformatics Analysis of the FUT2 Promoter

The Integrative Genomics Viewer (IGV) software (http://www.broadinstitute.org/igv/) was used to confirm the transcriptional start site (TSS) and the promoter region of *FUT2* genes based on our transcriptome sequencing of duodenal tissues of 18-resistant and -sensitive piglets. Moreover, the MethPrimer (http://www.urogene.org/cgi-bin/methprimer/methprimer.cgi) and Alibaba (http://www.gene-regulation.com/pub/programs/alibaba2/index.html?) software were used to analyze the CpG islands and transcription factor binding sites based on the sequence of the *FUT2* promoter region.

### 4.8. Methylation Sequencing of FUT2 Promoter

10 mg of jejunum and duodenum tissues from pigs were used to extract DNA using EpiTect bisulfite kit (Qiagen, Valencia, CA, USA). The PyroMark Assay Design software was used to design PCR primer sequence: forward, 5’-TTGAAGTAGGTATTTTTAGTTGGTGTAG-3’, reverse, 5’-CCATTCTACTAACTTACATCTCTCTTTTT-3’. DNA bisulfite conversion and bisulfite specific PCR, NGS library preparation and sequencing using the MiSeq platform (Illumina, San Diego, CA, USA), NGS data analysis, and digital methylation quantitation were performed according to the previous methods [19].

### 4.9. Electrophoretic Mobility Shift Assay

The *FUT2* wild-type (wt) (Forward primer: 5’-CATATGCCGCAGGAGCAGC, Reverse primer: GCTGCTCCTGCGGCATATG-3’) and *FUT2* mutant-type (mut) (Forward primer: 5’-CATATGTTTCAGGAGCAGC, Reverse primer: GCTGCTCCTGAAACATATG-3’) probes were generated based on the *FUT2* promoter region for Electrophoretic Mobility Shift Assays (EMSA). Antibody supershift assay Sp1 antibodies (CSB-PA298794) were obtained from Cusabio Biotech (Wuhan, China). The EMSA test was performed by the previous methods [19]

### 4.10. Statistical Analyses

Comparison between groups was made using Student’s t-test in the SPSS version 18.0 software (SPSS, Inc., Chicago, IL, USA). Each treatment had three repeats, and the results were presented as mean ± standard deviation (x ± SD). Differences were considered statistically significant with *p* < 0.05 or *p* < 0.01. A correlation analysis was performed pairwise for gene expression and methylation level by Pearson correlation.

## 5. Conclusions

Our study showed that the downregulation of *FUT2* correlates with *E. coli* F18 resistance. Furthermore, we found that the methylation of the mC-22 site residue in the *FUT2* promoter inhibited *FUT2* expression and that the Sp1 transcription factor might be regulated by methylation, suggesting that the methylation-induced inhibition of Sp1 binding to DNA reduces the transcriptional activity of the *FUT2* gene promoter, thereby improving the *E. coli* F18 resistance in piglets.

## Supplementary Materials

Supplementary materials can be found at http://www.mdpi.com/1422-0067/19/11/3301/s1.

## Abbreviations

*E. coli*	*Escherichia coli*
LPS	Lipopolysaccharide
PWD	Post-weaning diarrhea
FUT2	α(1,2) fucosyltransferase 2
Sp1	Specificity protein 1
EMSA	Electrophoretic mobility shift assays
NGS	Next-generation sequencing
RNA-seq	RNA sequencing
BSAS	Bisulfite amplicon sequencing
DEGs	Differential expression genes
TFBS	Transcription factor binding site
IGV	Integrative Genomics Viewer
TSS	Transcriptional start site
qRT-PCR	Quantitative real-time polymerase chain reaction
