# Supplementary material for: New Insight into the Molecular Mechanism of the FUT2 Regulating Escherichia coli F18 Resistance in Weaned Piglets
Source: Int J Mol Sci. 2018 Oct 24;19(11):3301. doi: 10.3390/ijms19113301 (PMC6275016; doi:10.3390/ijms19113301)
Supplement: Supplementary file 1 [file ijms-19-03301-s001.pdf]

Supplementary materials

Table S1. Real-time PCR primers and sequences

Gene	Accession number	Primer	Length
FUT2	U70881.2	F: 5′-AATCCCTGACCTCACTCCGTG-3′<br>R: 5′-CGGAACTACAACTGCTGGCC-3′	123 bp
TLR5	AB208697.2	F: 5′-GGTTCTCGCCCACCACATTA-3′<br>R: 5′-GGGTCCCAAAGAGTCGGAAG-3′	158 bp
FUT1	U70883	F: 5′-TTTTAAGCCCCCAAACTGCC-3′<br>R: 5′-TAAATCGACCCCATCAGCCTC-3′	126 bp
TAP2	NM_001206441.1	F: 5′-CCAACAGGTGGTTTTGGTCG-3′<br>R: 5′-AGAGTCCGTGCTCCATTTCG-3′	161 bp
IL-1β	NM_001005149	F: 5′- TGATTGTGGCAAAGGAGGA-3′<br>R: 5′- TTGGGTCATCATCACAGACG-3′	63 bp
FUT3	AF130972.1	F: 5′-CCCGAAGCCTTCATCCACAT-3′<br>R: 5′-CATCAAGGCCCAGCTGAAGA-3′	150 bp
GAPDH	NM_001206359.1	F: 5′-ACATCATCCCTGCTTCTACCGG-3′<br>R: 5′-CTCGGACGCCTGCTTCAC-3′	188 bp
β-actin	XM_003124280.3	F: 5′-TGGCGCCCAGCACGATGAAG-3′<br>R: 5′-GATGGAGGGGCCGGACTCGT-3′	149 bp

The selected genes were identified by real-time PCR. The housekeeping genes, *GAPDH* and *β-actin* were used as internal controls. The data were analyzed using the cycle threshold (C(t)) method.

Table S2. Differentially-expressed genes in duodenal tissues of individuals in the *E. coli* F18-resistant group compared with the sensitive group. Fold change means *E. coli* F18-resistant group/*E. coli* F18-sensitive group.

Overlapping DE genes from DESeq analyses	Associated ID	Length	log2(Foldchange)	p-value
XM_001924181.4	TUSC3	1201	0.635896	0.03195
XM_003125922.4	LOC100523668	1772	-1.0042	0.00125
XM_005661785.1	LOC100739218	611	-0.91693	0.04425
NM_214422.1	CYP3A39	299	0.943102	0.0056

XM_005668752.1	PYY	801	-1.6307	0.00175
XM_005667980.1	LOC100626755	1816	-1.1987	0.04825
XM_003133815.4	LOC100513317	812	-2.67131	0.0244
XM_005670219.1	LOC102161685	7406	-0.72903	0.04775
XM_003123974.4	EGR1	3165	-0.89788	0.0075
XM_005652369.1	FETUIN	1587	-3.32553	0.00195
NM_214362.1	LTF	2313	2.83955	5.00E-05
XM_005652468.1	LOC102160446	1847	-1.02299	0.0128
XM_001927551.2	IER3	1646	-0.83321	0.0294
NM_001097478.1	RPL14	710	-0.70401	0.033
XM_003357400.2	RAB7L1	2929	1.08917	0.03875
XM_003480608.2	SLC2A8	1976	0.756299	0.0203
NM_214069.1	FUT2	2316	-1.60013	0.00015
NM_214020.1	EGF	305	-1.45499	0.01095
XM_005661091.1	LOC733603	677	-4.54881	5.00E-05
NM_001115136.1	GPX2	799	0.800745	0.0288
XM_005657677.1	LOC102157588	1118	-1.80504	0.00195
XM_003130757.4	MYO3A	5549	1.03029	0.0063
XM_005653616.1	LOC102162205	5474	-1.17916	0.0045
XM_005656187.1	LOC100739514	1920	1.01733	0.04625
NM_214108.1	DPEP1	1462	0.856665	0.0061
NM_001243221.1	PSPH	1397	0.995417	0.00495
XM_005663709.1	GBP5	2414	-2.39837	5.00E-05
XM_003361626.1	LOC100621708	3423	-2.06353	5.00E-05
NM_001198926.1	GZMA	799	-0.73479	0.0358
NM_001195399.1	BMP2	1742	1.02939	0.0229
XM_003362105.2	LOC100624393	1718	-2.18768	0.001
XM_005655986.1	LOC100624036	1205	1.02585	0.0017
XM_005658955.1	APOBR	2269	-0.65463	0.04535
XM_003361092.2	LOC100620361	1571	-0.80853	0.01055
XM_005655072.1	LOC100522669	1471	-1.20156	0.00585
XM_001926804.3	P2RX7	2612	-0.65616	0.02845
XM_005671857.1	UPP2	2038	-0.55296	0.04955
NM_213931.1	ALOX12	2901	-1.692	0.0033
XM_005658535.1	LOC100627583	644	1.10196	0.03035
XM_005666261.1	LOC102167451	1038	0.794144	0.02015
XM_003483035.2	LOC100737474	3014	-0.60324	0.0437
XM_005660100.1	CER1	429	1.35886	0.02775
XM_005656792.1	LOC102167736	1163	-1.09057	0.0131
XM_005668016.1	SYT2	2253	1.37877	0.00065
XM_003128341.2	DAXX	2502	9.78833	0.0322
XM_005663716.1	GBP7	2615	-0.87118	0.0049
XM_005665463.1	PIK3R3	4133	1.71354	0.01835
XM_005658511.1	LOC100623504	1095	0.863126	0.01535

XM_005654402.1	EEF1A1	2029	0.130332	0.0432
NM_213754.2	LOC396596	737	inf	5.00E-05
XM_003481193.1	LOC100737346	818	-2.81719	5.00E-05
NM_001206441.1	TAP2	2617	-1.08738	0.0028
NM_001244884.1	AGXT2	2185	0.952083	0.02865
NM_214055.1	IL1B	1460	-1.23544	0.0291
XM_005657679.1	LOC102158041	1348	-1.63499	0.0447
NM_001038004.1	MMP9	2302	1.01043	0.0062
XM_003130633.4	LOC100515919	1371	-0.69166	0.0128
XM_003126503.2	LOC100523789	1249	-0.9264	0.0257
NM_001244695.1	PLA2G2D	1239	-1.13254	0.00325
XM_001925115.2	SH2D7	1863	0.778078	0.0418
XM_001928143.1	ELL3	1751	2.96172	5.00E-05
XM_001928587.2	LOC100156557	624	1.05082	0.0012
XM_003122320.4	LOC100521600	3234	-1.66162	0.0056
XM_001924824.2	TAAR1	1029	1.40054	0.03335
XM_005657681.1	LOC100620484	1625	-1.49804	0.00495
XM_003124230.2	IFITM1	780	0.675474	0.0289
XM_005662121.1	LOC100738647	2141	-0.68653	0.0349
NM_213978.1	B2M	514	1.87351	0.00015
XM_005654299.1	LOC102158723	1344	-0.80794	0.0354
XM_005667555.1	CR2	5252	5.18179	0.0025
XM_005662795.1	LOC100518658	926	-2.32212	5.00E-05
XM_005672404.1	LOC100620294	1772	-1.56716	0.0167
NM_001206402.1	TRIM31	2055	-1.25352	0.0002
XM_003483700.2	LOC100737264	3499	-2.8851	0.0014
XM_001929470.3	VPREB3	928	1.83299	0.00565
XM_005654355.1	SYNJ2	5842	0.71121	0.0178
XM_005667571.1	CR1	4119	0.823383	0.0375
NM_214317.1	AFP	2016	-1.057	0.00715
XM_005674257.1	LOC100737314	2169	1.13844	0.03265
NM_001008691.1	CXCL10	1129	-0.62659	0.03235
XM_003481432.2	BCAN	3328	1.06594	0.0191
XM_005658731.1	LOC102168053	396	-1.2757	0.0056
NM_001243873.1	LOC100511639	1370	-2.09626	0.00855
XM_003129101.1	CXCL13	1170	2.52731	5.00E-05
XM_003127961.4	LOC100524940	1913	0.596258	0.0457
XM_003357425.2	CR2	4124	3.0065	5.00E-05
XM_005661568.1	LOC102165335	3099	-1.3496	0.01655
XM_001926063.5	LOC100157318	2163	1.29673	0.0017
XM_003482521.2	GVIN1	6558	-0.84419	0.01515
XM_005660361.1	ZNF618	2868	-10.8995	0.04205
NM_213847.1	ICA	2315	-1.62107	0.00325
XM_005653499.1	LOC100736850	946	-0.85615	0.02175

XM_003355059.3	OPRK1	2116	-1.27642	0.048
NM_001243855.1	S1PR2	1778	1.1021	0.0172
XM_003133981.3	PLK2	2801	-0.84617	0.00785
NM_001136512.1	BCMO1	733	1.85939	5.00E-05
XM_005653435.1	LOC100525112	1481	0.807121	0.00735
NM_214412.1	CYP1A1	2860	-0.65399	0.0472
XM_003123279.2	LOC100524668	1296	-2.54178	0.04765
XM_005661808.1	LOC102165673	2296	-1.74491	0.01465
NM_001160080.1	DGAT2	336	0.948614	0.0179
NM_001123127.1	HSP70	293	1.08647	0.00955
XM_005673043.1	PCK1	2578	1.26342	0.02015
XM_005658540.1	LOC102161157	417	-2.02663	0.0001
XM_003123094.4	FUT3	1421	-1.4875	0.0008
XM_003354408.2	LOC100626247	690	3.39505	0.0056
XM_005655649.1	LOC102167487	3594	-3.03753	0.0002
XM_005652669.1	VNN1	2977	0.907864	0.0398
XM_005654962.1	CLK4	1848	-9.25354	0.03525
XM_001926306.5	LOC100156526	2308	0.89143	0.00855
XM_005669094.1	LOC100624611	2948	1.09271	0.0001
NM_001244733.1	INMT	969	-1.60548	0.00465
XM_001927885.4	PRAP1	603	0.803807	0.0054
XM_003130821.4	LOC100515572	986	-0.70649	0.0467
NM_001244729.1	C3H2orf40	743	1.771	0.00065
XM_005666877.1	LOC102159296	4150	-1.58056	0.0003
NM_214227.1	CD3E	1232	-0.66575	0.02655
NM_001244717.1	SLC13A2	2433	0.739653	0.01255
XM_005673835.1	ZCCHC16	1802	-1.28131	0.01385
XM_003361556.2	SLC2A5	1290	-0.56679	0.0444
NM_214215.2	FABP6	516	5.7094	0.0483
XM_005668209.1	LOC100516289	1872	-0.61927	0.03885
NM_001134824.1	CYP3A46	694	0.726046	0.01655
XM_005662708.1	LOC102166849	763	-2.71058	5.00E-05
NM_001243919.1	CUEDC1	2753	2.19684	0.0184
XM_003126977.2	LPCAT2	3375	-0.7163	0.0281
XM_003359590.2	SLC40A1	3499	-2.63403	0.00075
XM_003483081.1	LOC100738021	930	-1.45404	0.0276
XM_003480520.2	TMEM261	715	2.04124	0.0331
XM_005664953.1	LOC100736849	1690	-2.74382	5.00E-05
NM_214416.1	IYD	658	1.06405	0.01325
XM_005672303.1	LOC100739320	1053	1.14492	0.0044
XM_005662125.1	SOCS1	1250	-0.9659	0.0349
XM_005654032.1	LOC100738454	1683	1.40129	0.0043
NM_001243483.1	TFF3	784	0.96496	0.00245
NM_001123113.1	FOS	1143	-0.66785	0.0213

XM_005666775.1	CXCL11	1063	-1.02201	0.0195
XM_003482496.2	LOC100739437	1704	-1.19644	0.02295
XM_005668494.1	LOC100518213	2789	1.06456	0.0012
XM_001924902.3	LOC100153042	914	-1.29404	0.0423
NM_001243912.1	CD79B	1245	1.07636	0.0034
XM_003482441.2	SCLT1	1725	-0.72505	0.0497
XM_005662419.1	REG3G	801	2.04701	0.0053
XM_003356418.3	CTBS	728	1.76949	0.00275
XM_005658975.1	LOC100517370	1250	-3.33945	5.00E-05
XM_005658662.1	LOC102167956	612	-0.59211	0.03575
XM_003134585.4	GIMAP2	1382	-1.28529	0.0179
NM_001123158.1	PCK1	2591	1.09471	0.04365
XM_005669122.1	ITGAE	3493	-0.66584	0.0405
NM_213783.2	RETN	520	2.38509	0.00795
XM_003123966.2	LECT2	971	1.69756	0.00055
XM_005657599.1	BOK	999	0.761617	0.0461
NM_001244385.1	CDA	501	0.890004	0.0099
XM_003128013.4	SLC5A9	1840	0.755588	0.0288
XM_005654912.1	LOC102161910	273	#NAME?	0.0001
XM_003358091.2	PHOSPHO1	1872	1.3413	0.0307
XM_005671902.1	XIRP2	12126	-0.7761	0.032
XM_003484045.2	RP9	1302	0.725866	0.04615
XM_003123375.2	LOC100516246	533	2.04932	0.02865
NM_214110.1	SFTPD	1347	1.00122	0.01015
XM_005666529.1	LOC100624787	879	-1.75644	0.03985
XM_005658352.1	LOC100625274	1682	-2.6849	0.01505
XM_005663708.1	LOC102161784	1485	-1.02498	0.0106
NM_213876.1	AMCF-II	1489	-1.63322	0.00045
XM_005655795.1	LOC102167650	1245	-2.43708	5.00E-05
XM_005656775.1	LOC100525885	3349	-0.64522	0.0311
XM_003130159.3	DYNC1I1	2231	0.936887	0.0446
NM_001143710.1	GZMB	890	-0.82194	0.01615
XM_003128394.2	PGC	1523	0.825399	0.0095
XM_005667372.1	APOC3	574	0.773712	0.03415
XM_003482017.2	LOC100739434	2010	-2.96895	0.01145
NM_001001265.1	FCER1G	579	-0.72725	0.03775
XM_003134735.2	PTPRZ1	8006	0.863771	0.01895
NM_214420.1	CYP2C49	1958	0.83384	0.03645
XM_003123754.4	THBS4	2984	-1.14936	0.0231
XM_003357925.2	SLC16A3	2162	0.660299	0.02825
XM_003361314.3	LOC100621671	2196	0.785541	0.0264
XM_005666806.1	FGA	2123	-1.66832	0.0301
NM_213856.2	LCN1	172	#NAME?	0.00035
XM_003484284.1	LOC100737466	1874	-1.76125	0.00055

NM_214423.1	CYP3A29	536	0.719926	0.03355
XM_005661698.1	LOC100518542	1088	2.74388	0.0271
XM_003123638.4	GFPT2	3083	0.870829	0.0106
XM_003123159.3	C2H19orf59	1165	1.3897	0.0198
XM_003127418.4	LOC100522142	1979	-1.40782	0.00085
XM_001927980.4	TRIM31	2058	1.07417	0.00755
XM_005672658.1	LOC102165171	1427	1.54787	0.00075
XM_005659188.1	LOC100628107	2146	0.713564	0.03875
XM_005658277.1	LOC100623336	2673	-1.04453	0.0036
NM_001244642.1	LOC100156741	411	#NAME?	5.00E-05
XM_005670013.1	CCDC39	4118	0.844265	0.02
XM_005664534.1	LOC100524089	2899	-1.26632	0.02435
NM_001097450.1	C8A	1901	0.916473	0.0186
XM_005662048.1	CD19	2328	2.12344	0.00655
XM_005663706.1	GBP6	2539	-1.06801	0.0004
XM_003128317.4	LOC100512544	937	-1.37688	0.00095
XM_005655241.1	ABCG5	459	0.752098	0.03175
XM_003356781.3	ASB2	1834	-0.89386	0.04945
XM_005665062.1	PLA2G2A	1106	-4.06734	0.0218
XM_003359055.2	BLK	2097	1.13174	0.03385
XM_005667215.1	LOC100623257	1564	-2.89809	0.0005
NM_001101030.1	BPIFB2	1705	2.85326	0.00105
XM_005663979.1	PTPRR	2699	0.771148	0.015
NM_001114289.2	CXCL9	450	-1.88528	5.00E-05
XM_005660249.1	PAX5	2347	3.9528	5.00E-05
XM_005665757.1	LOC102166052	1584	-1.14727	0.0355
XM_005671469.1	AFAP1L2	3667	-0.60709	0.04865
XM_005655042.1	LOC100627004	1600	-1.65997	0.0297
XM_003131994.2	MYHC	6080	-0.74869	0.03325
XM_005658205.1	LOC100620987	1130	1.59877	0.0001
XM_003129240.4	LOC100513297	687	-1.50609	0.02375
NM_001123202.1	TLR5	2572	-0.72497	0.01525
NM_001278765.1	FFAR2-L	1105	1.01465	0.01115
NM_213776.1	CD2	1023	-0.7645	0.0072
XM_003124235.3	LOC100519082	1598	-0.75251	0.02025
XM_005672261.1	CCL20	633	1.38036	0.0026
XM_001928603.2	NUGGC	2936	1.31034	0.001
NM_213999.2	DUOX2	5712	1.07285	0.0003
XM_005655796.1	LOC102167769	694	-2.06739	0.0007
XM_003125609.4	MYBL1	4964	0.956325	0.00665
XM_003131161.2	ENPP7	2143	1.30432	0.03125
XM_005658532.1	LOC100627397	2126	-1.37145	0.00185
XM_005663244.1	LOC100152036	1134	-1.15553	0.03285
XM_005672371.1	NUTM2G	2417	-1.21502	0.03435

XM_005653274.1	LOC100525924	2731	-0.70478	0.03465
XM_005655794.1	LOC102167543	1333	-2.87869	0.00025
XM_005667971.1	LOC100517362	6178	-0.60595	0.04175
XM_003359855.2	SLC6A19	3383	0.988648	0.0034
NM_213852.1	MSMB	512	0.978307	0.0037
XM_003121158.2	ZC3H12D	2790	0.685929	0.03455
NM_001123196.1	SOCS3	590	-1.2684	0.01345
XM_005655946.1	LOC102158679	648	1.35088	0.0093
XM_003481515.2	LOC100737841	2351	-0.89188	0.00245
XM_003358448.2	SLC38A3	2445	1.24617	0.02435
NM_214442.2	PBD-2	347	1.26258	0.0271
XM_005668495.1	LOC102167556	1573	1.13836	0.00075
XM_003357321.2	TREH	1834	1.45365	5.00E-05
XM_005672695.1	LOC100152679	2562	0.927217	0.0015

Table S3. Gene ontology (GO) enrichment analysis of differentially expressed genes (DEGs) in duodenal tissues of individuals in the *E. coli* F18-resistant group compared with the sensitive group.

GO_accession	Description	Corrected p value	Gene_description
GO:0008009	chemokine activity	0.015022	CCL20, CXCL11, AMCF-II, CXCL10, CXCL9, CXCL13
GO:0042379	chemokine receptor binding	0.015022	CXCL11, CCL20, CXCL9, AMCF-II, CXCL10, CXCL13
GO:0004555	alpha,alpha-trehalase activity	0.015022	TREH, GBP7, LOC100620987
GO:0015927	trehalase activity	0.015022	TREH, LOC100620987, GBP7
GO:0002376	immune system process	0.015022	CD2, LOC100621671, AMCF-II, IL1B, CCL20, CXCL13, CXCL9, CXCL10, CXCL11, LOC100523668
GO:0005991	trehalose metabolic process	0.021547	TREH, LOC100620987, GBP7
GO:0005525	GTP binding	0.021547	PCK1, GIMAP2, GBP5, EEF1A1, GBP6, LOC100516289, GBP
GO:0032561	guanyl ribonucleotide binding	0.021547	TAP2, FUT2, LOC102161784
GO:0019001	guanyl nucleotide binding	0.021547	GBP5, TAP2, FUT2, PCK1, LOC102167556, RAB
GO:0005984	disaccharide metabolic process	0.021547	TREH, LOC100620987, GBP7
GO:0006955	immune response	0.021547	CCL20, AMCF-II, IL1B, CXCL13, CXCL11, CXCL10, CXCL9
GO:0001664	G-protein coupled	0.021547	CXCL13, AMCF-II, CXCL9,

	receptor binding		CXCL10, CXCL11, CCL20
GO:0009311	oligosaccharide metabolic process	0.027832	INMT, TREH, GBP7, LOC100620987
GO:0005125	cytokine activity	0.033923	CXCL11, CCL20, CXCL9, AMCF-II, CXCL10, CXCL13

Table S4. KEGG pathway analysis of differentially expressed genes (DEGs) in duodenal tissues of individuals in the *E. coli* F18-resistant group compared with the sensitive group.

Pathway_accession	ID	Corrected P-Value	KO_name
Linoleic acid metabolism	ssc00591	0.000000725	CYP3A39, ALOX12, 12-LOX, 15-LOX, ALOX15, PLA2G2D, CYP3A29, CYP2C49, CYP3A46, CYP3A88
Retinol metabolism	ssc00830	0.000961878	CYP3A29, CYP3A39, BCMO1, CYP2C49, CYP1A1, CYP3A46, CYP3A88
Arachidonic acid metabolism	ssc00590	0.001851731	GPX2, ALOX12, 12-LOX, 15-LOX, ALOX15, PLA2G2D, CYP2C49
Steroid hormone biosynthesis	ssc00140	0.004190856	CYP3A39, CYP3A29, CYP3A46, CYP3A88, CYP1A1, CYP2C49
Toll-like receptor signaling pathway	ssc04620	0.004613737	CXCL9, MIG, PIK3R3, CXCL10, TLR5, CXCL11, FOS, c-fos, IL1B
TNF signaling pathway	ssc04668	0.006785849	MMP9, PIK3R3, CXCL10, SOCS3, CCL20, FOS, c-fos, IL1B
Hematopoietic cell lineage	ssc04640	0.007840181	CD3E, CD3, CR1, CD2, CR2, CD19, IL1B
Chemical carcinogenesis	ssc05204	0.007889774	CYP3A39, CYP3A29, CYP3A46, CYP3A88, CYP1A1, CYP2C49
Legionellosis	ssc05134	0.010095999	TLR5, CR1, HSP70, HSP70B, HSPA6, EEF1A1, EF1A1, IL1B
Mineral absorption	ssc04978	0.0112216	ICA, PICA, SLC6A19
Antigen processing and presentation	ssc04612	0.012691938	TAP2, B2M, HSP70, HSP70B, HSPA6
B cell receptor signaling pathway	ssc04662	0.015705573	CD19, CD79B, FOS, c-fos, PIK3R3, CR2
Glycosphingolipid biosynthesis-lacto and neolacto series	ssc00601	0.017305544	FUT2, FUT3
Rheumatoid arthritis	ssc05323	0.031125286	CCL20, FOS, c-fos, IL1B, AMCF-II
Prion diseases	ssc05020	0.03293703	EGR1, C8A, IL1B
Primary immunodeficiency	ssc05340	0.040185407	TAP2, CD3E, CD3, CD19
Prolactin signaling pathway	ssc04917	0.04632882	SOCS3, FOS, c-fos PIK3R3, SOCS1

Pantothenate and CoA biosynthesis	ssc00770	0.054723531	VNN1
Chemokine signaling pathway	ssc04062	0.057017226	CXCL9, MIG, CXCL13, PIK3R, CXCL10, AMCF-II, CCL20, CXCL11
Complement and coagulation cascades	ssc04610	0.05915965	FGA, C8A, CR1, CR2

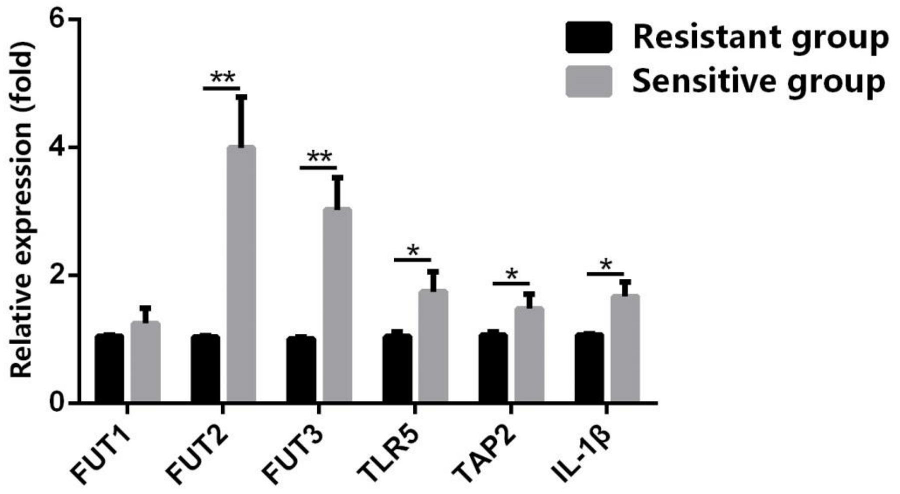

Figure S1. qRT-PCR Validation of selected differential expression genes (DEGs) from RNA-Seq expression profiles, *p < 0.05, **p < 0.01.
